# Risk of Venous Thromboembolism in Transgender People Undergoing Hormone Feminizing Therapy: A Prevalence Meta-Analysis and Meta-Regression Study

**DOI:** 10.3389/fendo.2021.741866

**Published:** 2021-11-09

**Authors:** Maria Totaro, Sara Palazzi, Chiara Castellini, Antonio Parisi, Federica D’Amato, Daniele Tienforti, Marco Giorgio Baroni, Sandro Francavilla, Arcangelo Barbonetti

**Affiliations:** ^1^ Andrology Unit, Department of Clinical Medicine, Public Health, Life and Environmental Sciences, University of L’Aquila, L’Aquila, Italy; ^2^ Neuroendocrinology and Metabolic Diseases, IRCCS Neuromed, Pozzilli (IS), Italy

**Keywords:** gender dysphoria, gender affirming hormone therapy, venous thomboembolism, transgender, estrogen

## Abstract

**Background:**

Although venous thromboembolism (VTE) is a recognized side effect of some formulations of estrogen therapy, its impact in transgender people remains uncertain. The aim of this study was to define pooled prevalence estimate and correlates of VTE in Assigned Males at Birth (AMAB) trans people undergoing gender affirming hormone therapy.

**Methods:**

A thorough search of MEDLINE, COCHRANE LIBRARY, SCOPUS and WEB OF SCIENCE databases was carried out to identify suitable studies. Quality of the articles was scored using the Assessment Tool for Prevalence Studies. Data were combined using random effects models and the between-study heterogeneity was assessed by the Cochrane’s Q and I^2^.

**Results:**

The eighteen studies included gave information about 11,542 AMAB undergoing gender affirming hormone therapy. The pooled prevalence of VTE was 2% (95%CI:1-3%), with a large heterogeneity (I^2 ^= 89.18%, P<0.0001). Trim-and-fill adjustment for publication bias produced a negligible effect on the pooled estimate. At the meta-regression analysis, a higher prevalence of VTE was significantly associated with an older age (S=0.0063; 95%CI:0.0022,0.0104, P=0.0027) and a longer length of estrogen therapy (S=0.0011; 95%CI:0.0006,0.0016, P<0.0001). When, according to the meta-regression results, the analysis was restricted to series with a mean age ≥37.5 years, the prevalence estimate for VTE increased up to 3% (95%CI:0-5%), but with persistence of a large heterogeneity (I^2 ^= 88,2%, P<0.0001); studies on younger participants (<37.5 years) collectively produced a pooled VTE prevalence estimate of 0% (95%CI:0-2%) with no heterogeneity (I^2^ = 0%, P=0.97). Prevalence estimate for VTE in series with a mean length of estrogen therapy ≥53 months was 1% (95%CI:0-3%), with persistent significant heterogeneity (I^2 ^= 84,8%, P=0.0006); studies on participants subjected to a shorter length of estrogen therapy (<53 months), collectively produced a pooled VTE prevalence estimate of 0% (95%CI:0-3%) with no heterogeneity (I^2 ^= 0%, P=0.76).

**Conclusions:**

The overall rate of VTE in AMAB trans people undergoing gender affirming hormone therapy was 2%. In AMAB population with <37.5 years undergoing estrogen therapy for less than 53 months, the risk of VTE appears to be negligible. Further studies are warranted to assess whether different types and administration routes of estrogen therapy could decrease the VTE risk in AMAB trans people over 37.5 years subjected to long-term therapy.

**Systematic Review Registration:**

[https://www.crd.york.ac.uk/PROSPERO/], identifier [CRD42021229916].

## Introduction

Transgender people do not experience gender as consistent with their birth sex. The non-correlation between experienced gender and biological sex, known as gender incongruence (1), can lead to stigma, depression, body uneasiness, social margination for Assigned Males at Birth (AMAB) and Assigned Females at Birth (AFAB) trans people (“gender dysphoria”). For those who want to change all/some physical features can start gender affirming care (hormonal and/or surgical treatment).

According to a meta-analysis by Collin and colleagues, the overall prevalence estimates for transgender diagnoses were 2.5 per 100,000 for AFAB and 5.8 per 100,000 for AMAB, although prevalence may vary based on different definitions (2).

World Professional Association for Transgender Health (WPATH) guidelines define hormone treatment as medically necessary for transgenders asking for medical interventions to affirm their gender (3). The Endocrine Society recommends oral, transdermal, or intramuscular 17β-estradiol for AMAB trans people (4). Oral administration includes micronized 17β-estradiol, conjugated estrogens and estradiol valerate, quickly cleaved to 17β-estradiol, while ethinyl estradiol is no longer recommend because of the poor safety profile (5). Co-administration with androgen inhibitors (cyproterone, spironolactone, progesterone) is often chosen to foster feminization (6, 7). However, the lack of international standardization of specific hormone regimens for gender affirming therapy in AMAB trans people hinders the knowledge of the side effects of hormone treatment, including venous thromboembolism (VTE).

The incidence rate of VTE in young women who do not use estrogens is about 1 in 10,000 per year (8–10). Much of the available data on thrombotic risk associated to estrogen treatment steams from studies on cisgender women treated with combined oral contraceptives (COCs), or with hormone replacement therapy (HRT) (11). In cisgender women, COC increases the risk of VTE by approximately 2–4 fold (10, 12) and a higher risk would result from increases in estrogen dose (13). Transdermal estrogen formulations used for HRT in postmenopausal women do not seem to be associated with a significant increase in the VTE risk (12, 14–16) and had showed a low thrombogenic profile in AMAB trans people, although there are no head-to-head studies with other estrogen formulations (17, 18). However, thrombophilia, smoking, obesity, age, major surgery and fractures are well-recognized risk factors in the general population and could contribute, alone or in combination, to promote VTE in COC and HRT users (19–22).

Results of studies on the thrombotic risk in cisgender women under COC or HRT should not be translated to AMAB trans people due to differences in age, estrogen formulations and doses (23); cisgender women and AMAB trans people do have genetic differences and AMAB usually also use androgen inhibitors. Furthermore, many data on the risk of VTE in AMAB trans people undergoing feminizing hormone treatment have been produced in case reports or small series, thus reaching a low statistical power (18). In a meta-analysis by Khan and colleagues (7), the incidence rate for VTE was 2.3 per 1,000 person-years (95% CI: 0.8 – 6.9). However, this estimate was burdened with a large and unexplained between-study heterogeneity (I ^2^ = 74%; P = 0.0039). More recently, a systematic review pointed to a significantly higher incidence of VTE in treated AMAB compared to AFAB trans people (42.8 *vs* 10.8 VTE per 10,000 patient years; P = 0.02) (24). Again, a large between-study heterogeneity arose, and the qualitative approach did not allow the identification of covariates potentially able to influence the pooled data.

Given the large unexplained heterogeneity among the studies, the actual impact and risk of VTE in AMAB trans people receiving gender affirming hormone therapy remains uncertain. In this light, we aimed to perform a systematic review and a meta-analysis of available studies to define pooled prevalence estimate and correlates of VTE in AMAB undergoing hormone feminizing therapy.

## Materials and Methods

The study was conducted according to the Preferred Reporting Items for Systematic Review and Meta-Analysis Protocols (PRISMA-P) (25). It also complies with the guidelines of Meta-Analyses and Systematic Reviews of Observational Studies (MOOSE) (26). The PRISMA-P and MOOSE checklists have been presented as **Supplementary Table 1** (A and B). The study is registered in the PROSPERO (International Prospective Register of Systematic Reviews) with the number CRD42021229916. (https://www.crd.york.ac.uk/PROSPERO/).

### Systematic Search Strategy

A systematic search was performed in MEDLINE, Scopus, Cochrane Library and Web of Science, including the following free and vocabulary terms: ‘gender dysphoria’, ‘gender identity’, ‘MTF’, ‘Assigned Males at Birth’, ‘AMAB’, ‘gender transition’, ‘transsexual’, ‘transfeminine’, ‘transwomen’, ‘gender affirmation’, ‘gender affirming hormone therapy’, ‘feminizing therapy’, ‘estrogen’, ‘hormone therapy’, ‘thrombosis’, ‘embolism’, ‘thromboembolism’, using the Boolean functions AND/OR. The search was restricted to English-language studies enrolling human participants, published up to April 2021. If it was not clear from the abstract whether the study contained relevant data, the full text was retrieved. The reference lists of the identified articles were also scrutinized to find possible additional pertinent studies.

### Inclusion and Exclusion Criteria

Eligible studies were identified according to a PECOS (Population, Exposure, Comparison/Comparator, Outcomes, Study design) model (**Supplementary Table 2**).

Studies were included in quantitative analysis if they reported the prevalence (or information for its calculation) of VTE events in AMAB trans people recruited from the general population or from cohorts of patients. Observational studies (case-control, cross-sectional, prospective and series of cases), as well as intervention studies were screened for eligibility. Only information about cases (AMAB trans people) were extracted from case-control studies. Duplicates were rigorously checked and removed.

Reviews, meta-analyses and studies lacking to assess the outcomes of interest were excluded. When the same population sample was used for multiple publications, only the study with the largest number of participants was included.

Two independent reviewers (M.T. and S.P.) evaluated the full text of all selected studies for eligibility, and, where disagreement occurred, a third reviewer (A.B.) took a decision after open discussion.

### Data Extraction

Data were extracted from the selected studies by four independent reviewers (A.P., S.P., F.D.A. and D.T.) by including the first author, publication year, country/geographic region, study design, the total number of AMAB and the number of those who have experienced a VTE event. When available, the mean age and body mass index (BMI) of the participants, smoking habit, type, dosage and duration of hormone therapy, diagnosis of comorbidities, including type-2 diabetes mellitus (T2DM), dyslipidemia and hypertension, were also taken into account. When summary statistics were not fully reported, these were calculated, whenever possible (27). Where data were missing, incomplete or inconsistent, the authors were contacted to obtain necessary information.

### Quality Assessment

Quality of the studies was assessed using an adapted Assessment Tool for Prevalence Studies (28). This tool, designed to assess the risk of bias in prevalence studies, considers 10 different items, including representativeness and selection of the study population, likelihood of non-response bias, process of data collection, appropriateness of the definition of cases (AMAB trans people) as well as of the measurement of the parameter of interest (prevalence of VTE). Response options for individual items were either low or high risk of bias and a summary assessment of the overall risk of bias was based on the subjective judgment attributed to the 10 items: 7-10 items with ‘low risk’ judgment indicated an overall low risk of bias; 4-6 items with ‘low risk’ judgment indicated an overall moderate risk of bias; 0-3 items with ‘low risk’ judgment indicated an overall high risk of bias.

Quality assessment was performed independently by 2 reviewers (M.T. and C.C.) and any disagreement was resolved by involving a third reviewer (A.B.) who re-evaluated the original study.

### Statistical Analysis

The pooled prevalence of VTE was estimated by a random-effects model which assumes that the included studies have varying effect sizes, thus providing a conservative estimate of the overall effect. The 95% confidence intervals (CIs) of the prevalence reported in individual studies were estimated from the proportion of cases of VTE and the sample size, using the binomial Clopper-Pearson exact method. After ascertaining the non-normal distribution of the original data sets (by the Shapiro-Wilk test), the Freeman-Tukey double arcsine transformation was applied to the primary study data to approximate normality. The final pooled results and 95% CIs were back transformed and expressed as percentages for an easier interpretation. An inverse variance method was used for weighting each study in the pooled estimates. The Cochran’s Chi square (Cochran’s Q) test and the I^2^ test were used to analyze the statistical heterogeneity between the results of different studies: an I^2^ >50% and/or P <0.05 indicated substantial heterogeneity (29).

Publication bias was assessed through funnel plots (30) and the Begg adjusted rank correlation test (31). In case of asymmetric funnel shape, Duval and Tweedie’s ‘trim-and-fill’ analysis was carried out to detect putative missing studies which could rebalance the distribution; the analysis provides an adjusted pooled estimate taking the additional studies into account, thus correcting the analysis for publication bias (32).

Covariates that could affect the estimates, such as the mean age and BMI of the participants, comorbidities, smoking habit, type, dosage and duration of hormone therapy were included in linear meta-regression models.

Data were analyzed and graphed using the packages ‘metafor’ and ‘ggplot2’ of the R statistical software (version 4.0.3, 2020; The R Foundation for Statistical Computing, Vienna, Austria).

## Results

### Study Selection and Quality Assessment

**Figure 1:**
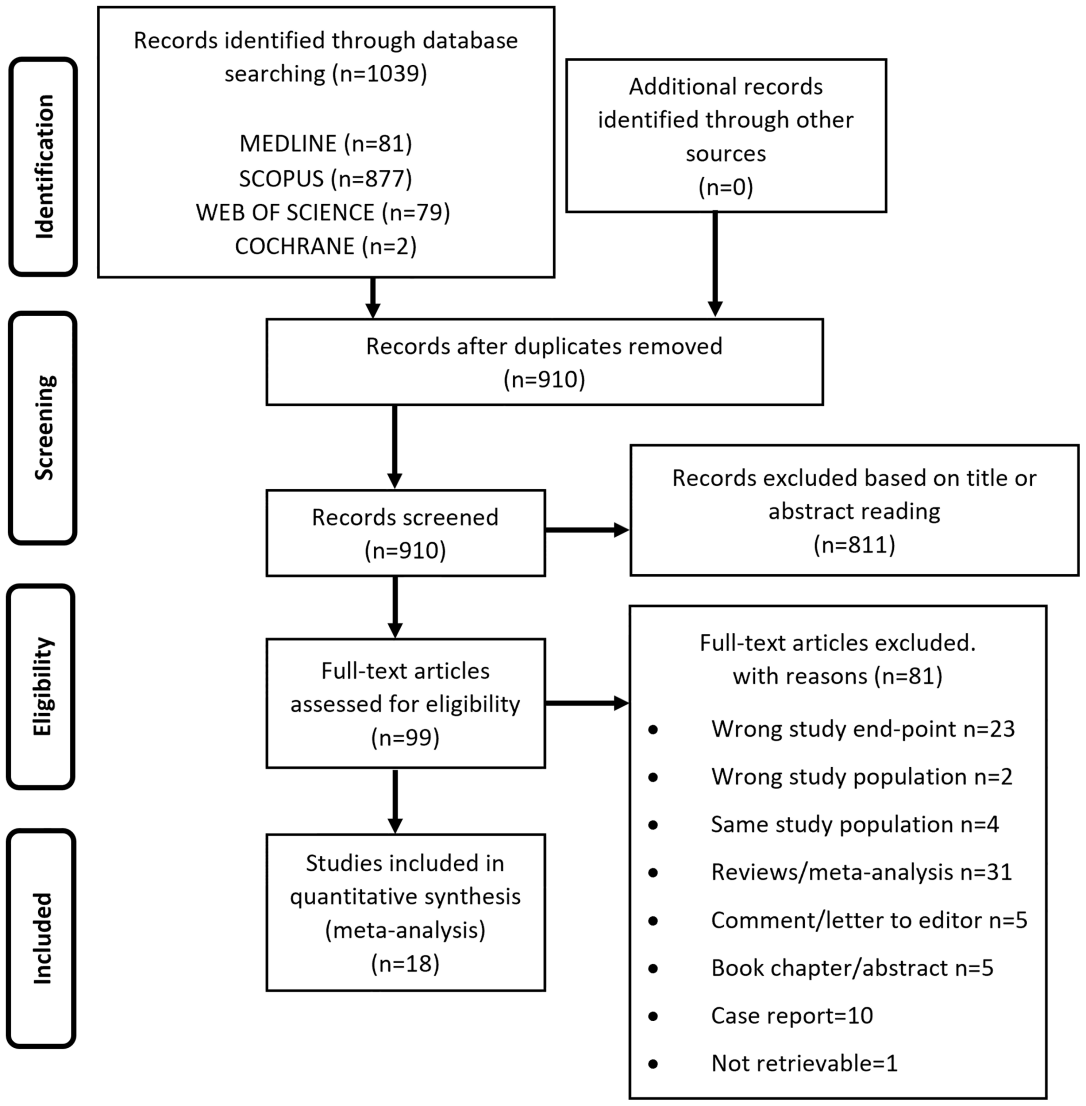
Flow diagram showing an overview of the study selection process.

From the electronic search we retrieved a total of 1039 studies and, after removal of duplicates, 910 studies were left. 811 papers were excluded as irrelevant, based on title and abstract reading. Hence, as shown in **Figure 1**, 99 studies were identified, of which 18 met the inclusion criteria (17, 33–49). The studies by Asscheman and colleagues published in 1989 (50) and 2011 (51) were excluded since the population under investigation was already included in the paper by van Kesteren et al., 1997 (47). The studies by Wierckx et al., 2012 (52) and Wierckx et al., 2014 (53) were also excluded because subjects included were considered in the study by Wierckx et al., 2013 (48). Details of the selected articles are summarized in **Table 1** and **Supplementary Table 3**.

**Table 1:** Characteristics of the included studies.

Study	AMAB (n)	VTE (n, %)	Thrombophilia inherited risk factors	Months of therapy (mean)	Mean age (years)	Mean BMI (kg/m^2^)	Oral estrogen (n, %)	DM (n, %)	Hypertension (n, %)	Dyslipidemia (n, %)	Current smokers (n, %)	Estrogen valerate (n, %)
Arnold et al. (33)	676	1 (0.15%)	Case with VTE negative for anti-phospholipid Abs, factor V Leiden or PT gene mutations	22.8	33.2	26.6	676 (100%)	43 (6.4%)	88 (13.0%)	59 (8.7%)	143 (21.2%)	0 (0.0%)
Dittrich et al. (34)	60	1 (1.67%)	Case with VTE positive for a homozygous mutation in C677 T MTHFR	24.0	38.4	24.2	60 (100%)	0 (0.0%)	0 (0.0%)	0 (0.0%)	NR	60 (100%)
Getahun et al. (35)	2842	61 (2.15%)	NR	NR	NR	NR	853 (30.0%)	NR	461 (16.0%)	170 (6.0%)	434 (15.0%)	NR
Jain et al. (36)	92	0 (0.00%)	NR	40.8	31.0	NR	0 (0.0%)	0 (0.0%)	NR	NR	NR	0 (0.0%)
Kozato et al. (37)	662	1 (0.20%)	NR	NR	35.6	25.7	210 (31.7%)	NR	NR	NR	NR	0 (0.0%)
Meyer et al. (38)	155	3 (1.90%)	One case with VTE positive for heterozygous PT mutation	NR	NR	NR	73 (47.1%)	NR	NR	NR	NR	155 (100%)
Mullins et al. (39)	182	0 (0.00%)	Thrombophilia screening: elevated PAI-1 levels, n = 5; PAI-1 gene polymorphism, n = 5; elevated factor VIII level, n = 4	20.8	18.0	NR	165 (90.7%)	NR	NR	NR	94 (51.6%)	0 (0.0%)
Nolan et al. (40)	178	1 (0.60%)	NR	67.2	36.2	25.2	NR	8 (4.5%)	15 (8.4%)	12 (6.7%)	NR	NR
Nota et al. (41)	2517	73 (2.90%)	NR	NR	NR	NR	NR	NR	NR	NR	NR	NR
Ott et al. (17)	162	0 (0.00%)	Thrombophilia screening: aPC resistance, n = 12; aPC resistance + homozygous factor V Leiden mutation, n = 1	64.8	36.6	22.7	0 (0.0%)	2 (1.2%)	35 (21.6%)	62 (38.3%)	96 (59.3%)	0 (0.0%)
Prior et al. (42)	50	0 (0.00%)	NR	12.0	32.7	NR	50 (100%)	NR	NR	NR	NR	0 (0.0%)
Pyra et al. (43)	2509	19 (0.80%)	NR	53.1	37.5	32.9	946 (37.7%)	83 (3.3%)	49 (1.9%)	NR	NR	NR
Schlatterer et al. (44)	46	0 (0.00%)	NR	NR	NR	NR	26 (56.5%)	NR	NR	NR	20 (43.5%)	0 (0.0%)
Seal et al. (45)	330	4 (1.20%)	NR	109.2	45.6	NR	330 (100%)	1 (0.3%)	2.6%	NR	NR	163 (49.4%)
Tack et al. (46)	21	0 (0.00%)	NR	15.6	17.6	NR	21 (100%)	NR	NR	NR	NR	21 (100%)
van Kesteren et al. (47)	816	45 (5.50%)	NR	NR	46.5	NR	NR	NR	61 (7.5%)	NR	NR	0 (0.0%)
Wierckx et al. (48)	214	11 (5.10%)	NR	152.0	43.7	24.7	99 (46.3%)	8 (3.7%)	NR	NR	NR	91 (42.5%)
Wilson et al. (49)	30	0 (0.00%)	NR	6.0	38.6	NR	23 (76.7%)	0 (0.0%)	NR	NR	2 (6.6%)	0 (0.0%)

Values are presented as mean or number (%). Abs, antibodies; AMAB, Assigned Males at Birth; BMI, body mass index; DM, diabetes mellitus; MTHFR, methylenetetrahydrofolate reductase; NR, not reported; PAI-1, plasminogen activator inhibitor-1; PT, prothrombin; VTE, thromboembolic events.

**Table 2** showed quality assessment of the studies: 15 studies were considered at low/moderate risk of bias, whereas an overall high risk of bias was attributed to the remaining 3 studies.

**Table 2:** Quality assessment of the included studies.

Study	Q1	Q2	Q3	Q4	Q5	Q6	Q7	Q8	Q9	Q10	OVERALL
**Arnold et al.** (33)	L	L	H	H	L	L	L	L	L	L	Low risk of bias
**Dittrich et al.** (34)	H	H	H	L	L	L	L	L	L	L	Low risk of bias
**Getahun et al.** (35)	L	H	H	L	H	H	L	H	L	H	Moderate risk of bias
**Jain et al.** (36)	H	H	H	L	L	L	L	L	L	L	Low risk of bias
**Kozato et al.** (37)	H	H	H	H	H	L	H	L	L	H	High risk of bias
**Meyer et al.** (38)	H	H	H	H	H	L	L	L	L	L	Moderate risk of bias
**Mullins et al.** (39)	L	H	H	L	H	L	L	H	H	L	Moderate risk of bias
**Nolan et al.** (40)	H	H	H	L	H	L	L	L	L	H	Moderate risk of bias
**Nota et al.** (41)	H	H	H	H	H	L	L	L	L	L	Moderate risk of bias
**Ott et al.** (17)	H	H	H	H	H	L	L	L	L	L	Moderate risk of bias
**Prior et al.** (42)	H	L	H	L	H	L	L	L	L	L	Low risk of bias
**Pyra et al.** (43)	L	H	H	L	H	L	H	L	H	H	Moderate risk of bias
**Schlatterer et al.** (44)	H	H	H	H	L	L	L	H	H	L	Moderate risk of bias
**Seal et al.** (45)	H	H	H	H	H	H	L	H	L	L	High risk of bias
**Tack et al.** (46)	H	H	H	H	L	L	H	L	H	H	High risk of bias
**van Kesteren et al.** (47)	L	L	H	L	H	L	L	L	L	L	Low risk of bias
**Wierckx et al.** (48)	H	H	H	H	L	L	H	L	H	H	Moderate risk of bias
**Wilson et al.** (49)	H	L	H	H	L	L	L	L	L	L	Low risk of bias

H, High risk; L, Low risk.

Q1. Was the study’s target population a close representation of the national population in relation to relevant variables?

Q2. Was the sampling frame a true or close representation of the target population?

Q3. Was some form of random selection used to select the sample, OR was a census undertaken?

Q4. Was the likelihood of non-response bias minimal?

Q5. Were data collected directly from the subjects (as opposed to a proxy)?

Q6. Was an acceptable case definition used in the study?

Q7. Was the study instrument that measured the parameter of interest (prevalence of thromboembolic events) shown to have reliability and validity?

Q8. Was the same mode of data collection used for all subjects?

Q9. Was the length of the shortest prevalence period for the parameter of interest appropriate?

Q10. Were the numerator(s) and denominator(s) for the parameter of interest appropriate?

OVERALL. Summary item on the overall risk of study bias: 7-10 items with ‘low risk’ judgment = overall low risk of bias; 4-6 items with ‘low risk’ judgment = overall moderate risk of bias; 0-3 items with ‘low risk’ judgment = overall high risk of bias.

### Synthesis of Results and Publication Bias

**Figure 2:**
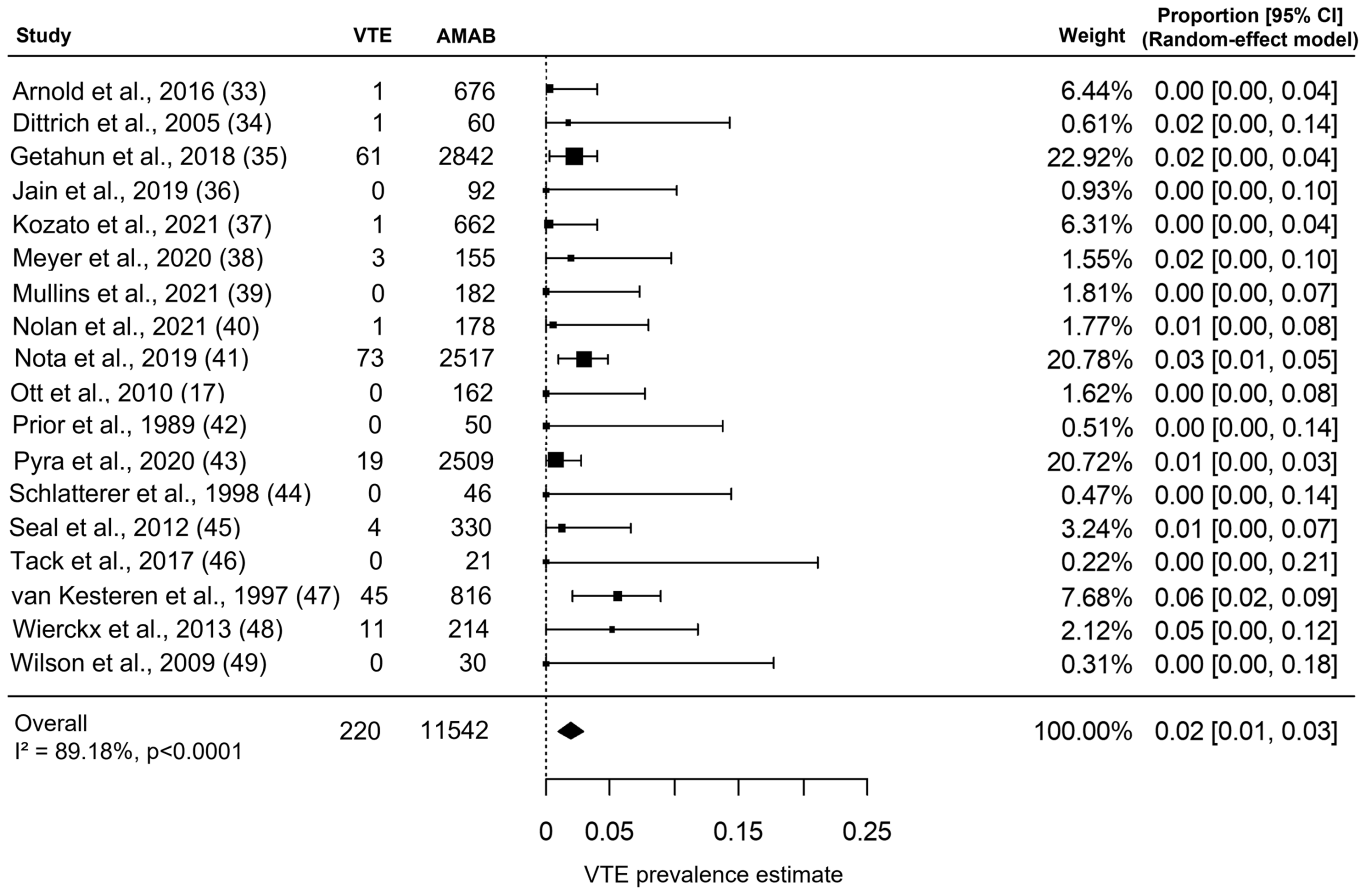
Forest plot depicting the pooled prevalence estimate for venous thromboembolism (VTE) in Assigned Males at Birth (AMAB) trans people. Diamond indicates the overall summary estimate and the width of the diamond represents the 95% confidence interval (CI); boxes indicate the weight of individual studies in the pooled result.

As shown in **Figure 2**, the included studies collectively gave information about VTE in 11,542 AMAB trans people, resulting in a pooled VTE prevalence estimate of 2.0% (95% CI: 1.0 - 3.0%), with a large heterogeneity (I^2^ = 89.2%, *P*_for heterogeneity_ <0.0001).

Although the Begg’s rank correlation test suggested a not significant asymmetry in funnel plot of VTE (Kendall’s τ = 0.072, P = 0.71), the trim-and-fill analysis identified five putative ‘missing study’ on the right side of distribution (**Supplementary Figure 1**). Nevertheless, when the funnel plot distribution was rebalanced by including these putative additional studies, the adjustment for publication bias produced a negligible effect on the pooled prevalence estimate for VTE (adjusted pooled prevalence: 1.9%, 95% CI: 1.0 - 2.9%).

### Analysis of the Between-Study Heterogeneity: Meta-Regression and Sub-Group Analyses

Meta-regression analyses were carried out to find out covariates that could affect the prevalence estimate. No significant relationship with VTE was found for BMI (S = -0.0021; 95% CI: -0.0199, 0.0158, P = 0.8), number of current smokers (S = -0.0017; 95% CI: -0.0041, 0.0007, P = 0.2), number of participants taking oral estrogen therapy (S = 0.0000; 95%CI: -0.0010, 0.0010, P = 0.9), number of participants taking estrogen valerate (S = 0.0008; 95% CI: -0.0003, 0.0019, P = 0.2), diagnosis of T2DM (S = -0.0018; 95% CI: -0.0220, 0.0183, P = 0.9), dyslipidemia (S = -0.0024; 95% CI: -0.0063, 0.0014, P = 0.2), and hypertension (S = -0.0029; 95% CI: -0.0103, 0.0044, P = 0.4).

**Figure 3:**
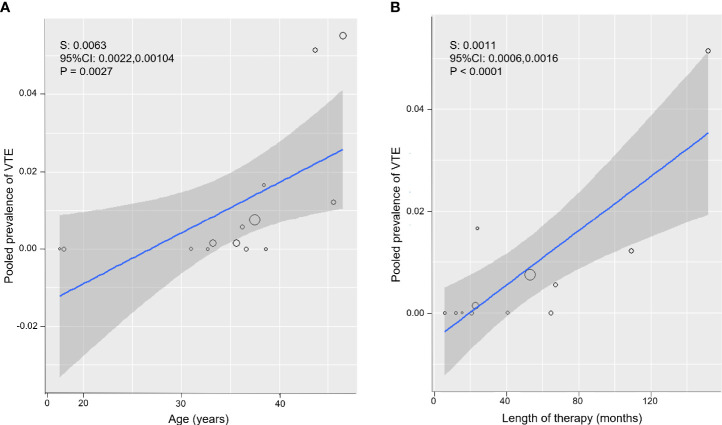
Meta-regression bubble plots: prevalence of venous thromboembolism (VTE) in Assigned Males at Birth (AMAB) trans people as a function of the mean age **(A)** and mean length of estrogen therapy **(B)**. The predicted effects (solid line) with corresponding confidence intervals (gray range) are also shown. CI, confidence interval; S, slope.

Both an older age of the participants and a longer length of estrogen therapy were significantly associated with a higher prevalence of VTE (for mean age of participants: S = 0.0063; 95% CI: 0.0022, 0.0104, P = 0.0027, **Figure 3A**; for mean months of estrogen therapy: S = 0.0011; 95% CI: 0.0006, 0.0016, P <0.0001, **Figure 3B**).

To substantiate the impact of the age and therapy duration as sources of the between-study heterogeneity, in subsequent sub-group analyses, pooled estimates were calculated separately for studies enrolling AMAB trans people below and above 37.5 years of age and for those on participants under estrogen therapy for less or more than 53 months. Dichotomization values were chosen according to the distributions of meta-regression bubble plots (**Figure 3**).

**Figure 4:**
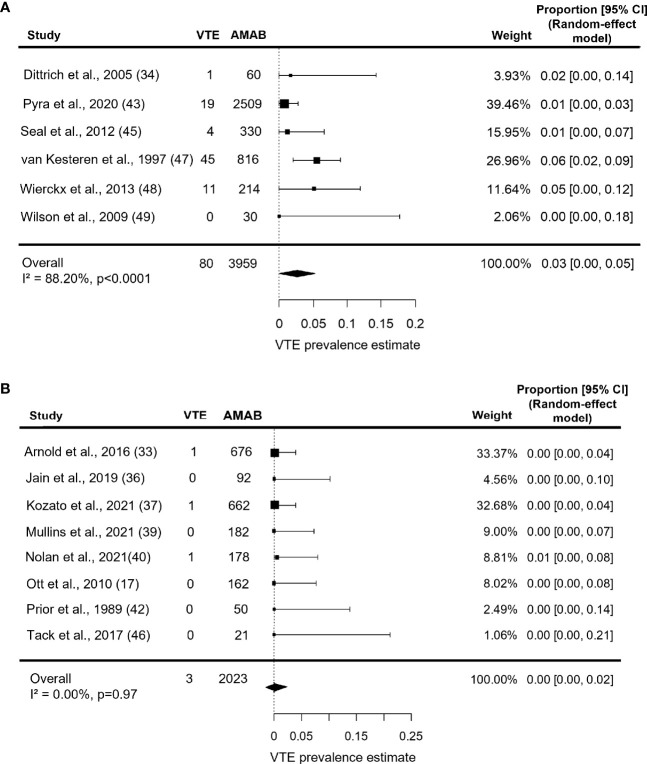
Forest plots depicting the results of the subgroup analysis of the prevalence of venous thromboembolism (VTE) in Assigned Males at Birth (AMAB) trans people by mean age. The pooled prevalence estimate was calculated separately for studies enrolling AMAB **(A)** above and **(B)** below 37.5 years of age. Diamonds indicate the overall summary estimates and width of the diamonds represents the 95% confidence interval (CI); boxes indicate the weight of individual studies in the pooled results.

When analysis was restricted to series with a mean age ≥37.5 years, the prevalence estimate for VTE increased up to 3.0% (95% CI: 0.0 - 5.0%), but with persistence of a large heterogeneity (I^2^ = 88.2%, P < 0.0001; **Figure 4A**). On the contrary, studies on younger participants (mean age <37.5 years) collectively produced a pooled VTE prevalence estimate of 0.0% (95% CI: 0.0 - 2.0%) with no heterogeneity (I^2^ = 0.0%, P = 0.97; **Figure 4B**).

**Figure 5:**
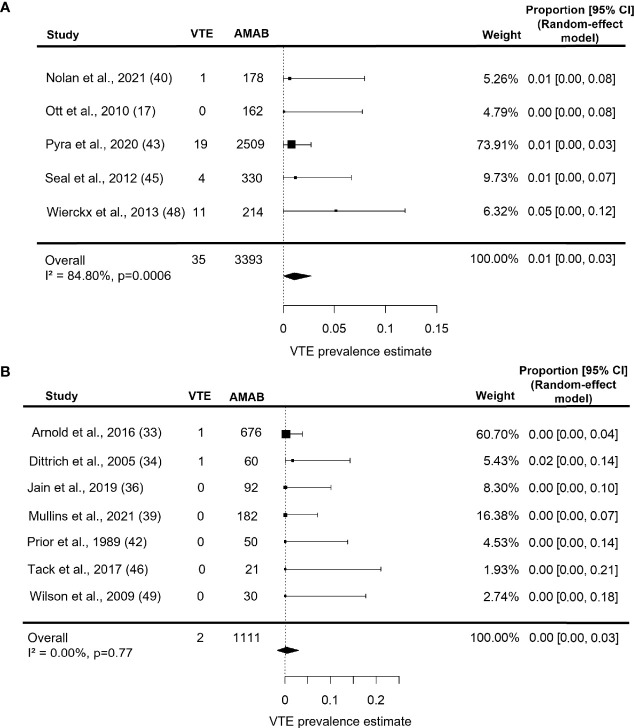
Forest plots depicting the results of the subgroup analysis of the prevalence of venous thromboembolism (VTE) in Assigned Males at Birth (AMAB) trans people by length of estrogen therapy. The pooled prevalence estimate was calculated separately for studies enrolling AMAB under estrogen therapy for **(A)** more and **(B)** less than 53 months. Diamonds indicate the overall summary estimates and width of the diamonds represents the 95% confidence interval (CI); boxes indicate the weight of individual studies in the pooled results.

Estimate prevalence for VTE in series under estrogen therapy for more than 53 months was 1.0% (95% CI: 0.0 - 3.0%), with persistent significant heterogeneity (I^2^ = 84.8%, P = 0.0006, **Figure 5A**). As shown in **Figure 5B**, studies on participants under estrogen therapy for less than 53 months, instead, produced a pooled VTE prevalence estimate of 0.0% (95% CI: 0.0 - 3.0%) with no heterogeneity (I^2^ = 0.0%, P = 0.77).

## Discussion

To our knowledge, this is the largest meta-analysis performed on VTE risk in AMAB trans people under gender-affirming hormone therapy: it included 18 studies, collectively giving information about 11,542 AMAB trans people. The overall pooled VTE prevalence estimate was 2%, but with large heterogeneity. Our results were similar to those of Khan and colleagues (7), who reported an incidence rate for VTE in AMAB treated with estrogens of 2.3 per 1,000 person-years, resulting from the analysis of a smaller number of studies. According to a recent systematic review by Kotamarti et al. (24), the incidence of VTE in AMAB would be significantly higher than in AFAB trans people.

A number of factors could contribute to the variable VTE risk in transgender people undergoing gender affirming treatment, including the type of estrogen and the route of administration, age at the estrogen therapy onset, length of therapy, concomitant conditions such as smoking, obesity, thrombophilia and comorbidities (16, 20–22, 54–56). In the present study, meta-regression analyses showed no significant relationship of VTE with BMI, smoking, diagnosis of T2DM, dyslipidemia and hypertension, albeit with the due caution this subject deserves due to the lack of information about these variables in many studies (**Table 1** and **Supplementary Table 3**). Indeed, it is known that obesity increases the risk of VTE in cisgender women using COCs (21) and the combination of COCs and smoking could exert a synergistic effect (22). Interestingly, consistent with our findings, in the recent systematic review by Kotamarty et al. (24), although AMAB trans people exhibited a lower BMI and an almost 2-fold higher prevalence of smoking compared to cisgender women, these variables were not correlated with the risk of VTE. Moreover, in the present meta-analysis, neither estrogen valerate nor oral estrogen use was related to VTE risk. Unfortunately, the dearth of information about type of estrogen and route of administration (**Supplementary Table 3**) did not allow us to carry out sub-group analyses with these variables.

Worth noting and previously not reported was the here revealed significant association of VTE with an older age of participants and a longer length of estrogen therapy, thus indicating that the longer the exposure time to therapy, the greater the rate of thromboembolic complications for AMAB trans people. Accordingly, when we restricted the analyses to younger series (mean age <37.5 years) and those under estrogen therapy for less than 53 months, the risk was wiped out with no between-study heterogeneity. Therefore, we surmise that the enrollment of series with different mean age and different length of estrogen therapy contributed to the large between-study heterogeneity. Although statistical analyses produced an overall VTE prevalence of 0% in sub-groups younger than 37.5 years and under estrogen therapy for less than 53 months, a complete absence of risk would be unrealistic in these populations. Our results should instead suggest that a not negligible rate of VTE, albeit low, has to be taken into account after 4-5 years of estrogen therapy, especially in older AMAB trans people. This latter finding, if on one hand could be due to an actual thrombophilic effect of prolonged estrogen treatment regimens (54), on the other, it could reflect a higher number of VTE diagnoses arising over time from regular control visits which usually trans people undergo in clinical settings. The persistence of a large between-study heterogeneity in these sub-analyses might be related to the different prevalence of comorbidities in the study populations, as well as to other variables, including the possible influence of different regimens of antiandrogens.

This study has some limitations. First, as mentioned above, the paucity of available data did not allow to perform reliable quantitative analyses to assess the best estrogen treatment regimen associated with the lowest risk for VTE in this population. However, transdermal estrogens and oral estradiol valerate are recommended by the current WPATH guidelines in people with VTE risk factors (3) while the use of ethinyl estradiol formulation is not recommended (18). Furthermore, the impact of inherited risk factors for VTE in AMAB trans people undergoing gender affirming hormone therapy remains uncertain as only five studies reported results of thrombophilia screening (**Table 1**). Finally, the trim-and-fill analysis revealed a possible publication bias, suggesting that published studies might be not fully a representative sample of the available evidence. Nevertheless, the corrected pooled OR, taking into account the putative missing studies, demonstrated that publication bias did not substantially affect the overall estimate.

In conclusion, the overall rate of VTE in AMAB trans people undergoing gender-affirming hormone therapy was 2%. Our analyses revealed that in AMAB series with younger age (<37.5 years) and under estrogen therapy for less than 53 months the risk of VTE appears to be negligible. Further studies investigating type and modalities of estrogen therapy are warranted to better manage the risk of VTE in this population.

## Data Availability Statement

The raw data supporting the conclusions of this article will be made available by the authors, without undue reservation.

## Author Contributions

MT and AB conceived the concept and design. MT, SP, and AB evaluated the full text of all selected studies for eligibility. AP, SP, FD’A, and DT were involved in the acquisition of the data. MT, AB, and CC were involved in evaluation of quality assessment. MT and AB were involved in the statistical analysis and interpretation of the data. MT wrote the article under SF supervision. MB and SF critically reviewed the article. All authors contributed to the article and approved the submitted version.

## Funding

This research was supported by the Ministero dell’Istruzione, Università e Ricerca (MIUR), grant number: 2017XLFJAX.

## Conflict of Interest

The authors declare that the research was conducted in the absence of any commercial or financial relationships that could be construed as a potential conflict of interest.

## Publisher’s Note

All claims expressed in this article are solely those of the authors and do not necessarily represent those of their affiliated organizations, or those of the publisher, the editors and the reviewers. Any product that may be evaluated in this article, or claim that may be made by its manufacturer, is not guaranteed or endorsed by the publisher.

## Supplementary Material

Supplementary Figure 1 Funnel plots of results from studies assessing the prevalence of venous thromboembolism (VTE). The trim-and-fill analysis identified five putative missing studies (white circle) on the right side of distribution.

## References

[1] Dakić T . New Perspectives on Transgender Health in the Forthcoming 11th Revision of the International Statistical Classification of Diseases and Related Health Problems: An Overview of Gender Incongruence - Depathologization, Considerations and Recommendations for Practitioners. Psychiatr Danub (2020) 32(2):145–50. doi: 10.24869/psyd.2020.145 32796778

[2] Collin L Reisner SL Tangpricha V Goodman M . Prevalence of Transgender Depends on the “Case” Definition: A Systematic Review. J Sex Med (2016) 13(4):613–26. doi: 10.1016/j.jsxm.2016.02.001 PMC4823815 27045261

[3] Coleman E Bockting W Botzer M Cohen-Kettenis P DeCuypere G Feldman J . Standards of Care for the Health of Transsexual, Transgender, and Gendernonconforming People, Version 7. Int J Transgenderism (2012) 13:165–232. doi: 10.1080/15532739.2011.700873

[4] Hembree WC Cohen-Kettenis PT Gooren L Hannema SE Meyer WJ Murad MH . Endocrine Treatment of Gender-Dysphoric/Gender-Incongruent Persons: An Endocrine Society Clinical Practice Guideline. J Clin Endocrinol Metab (2017) 102(11):3869–903. doi: 10.1210/jc.2017-01658 28945902

[5] Asscheman H T’Sjoen G Lemaire A Mas M Meriggiola MC Mueller A . Venous Thrombo-Embolism as a Complication of Cross-Sex Hormone Treatment of Male-to-Female Transsexual Subjects: A Review. Andrologia (2014) 46(7):791–5. doi: 10.1111/and.12150 23944849

[6] Tangpricha V den Heijer M . Oestrogen and Antiandrogen Therapy for Transgender Women. Lancet Diabetes Endocrinol (2017) 5(4):291–300. doi: 10.1016/S2213-8587(16)30319-9 27916515 PMC5366074

[7] Khan J Schmidt RL Spittal MJ Goldstein Z Smock KJ Greene DN . Venous Thrombotic Risk in Transgender Women Undergoing Estrogen Therapy: A Systematic Review and Metaanalysis. Clin Chem (2019) 65(1):57–66. doi: 10.1373/clinchem.2018.288316 30602475

[8] Samuelsson E Hägg S . Incidence of Venous Thromboembolism in Young Swedish Women and Possibly Preventable Cases Among Combined Oral Contraceptive Users. Acta Obstet Gynecol Scand (2004) 83(7):674–81. doi: 10.1111/j.0001-6349.2004.00574.x 15225194

[9] Lidegaard Ø Nielsen LH Skovlund CW Skjeldestad FE Lokkegaard E . Risk of Venous Thromboembolism From Use of Oral Contraceptives Containing Different Progestogens and Oestrogen Doses: Danish Cohort Study, 2001-9. BMJ (2011) 343:d6423. doi: 10.1136/bmj.d6423 22027398 PMC3202015

[10] de Bastos M Stegeman BH Rosendaal FR Van Hylckama Vlieg A Helmerhorst FM Stijnen T . Combined Oral Contraceptives: Venous Thrombosis. Cochrane Database Syst Rev (2014) 3):CD010813. doi: 10.1002/14651858.CD010813.pub2 PMC10637279 24590565

[11] Skeith L Le Gal G Rodger MA . Oral Contraceptives and Hormone Replacement Therapy: How Strong a Risk Factor for Venous Thromboembolism? Thromb Res (2021) 202:134–8. doi: 10.1016/j.thromres.2021.03.012 33836493

[12] Canonico M Oger E Plu-Bureau G Conard J Meyer G Lévesque H . Estrogen and Thromboembolism Risk (ESTHER) Study Group. Hormone Therapy and Venous Thromboembolism Among Postmenopausal Women: Impact of the Route of Estrogen Administration and Progestogens: The ESTHER Study. Circulation (2007) 115(7):840–5. doi: 10.1161/CIRCULATIONAHA.106.642280 17309934

[13] Practice Committee of the American Society for Reproductive Medicine . Electronic Address: ASRM@asrm.org; Practice Committee of the American Society for Reproductive Medicine. Combined Hormonal Contraception and the Risk of Venous Thromboembolism: A Guideline. Fertil Steril (2017) 107(1):43–51. doi: 10.1016/j.fertnstert.2016.09.027 27793376

[14] Lidegaard Ø Nielsen LH Skovlund CW Lokkegaard E . Venous Thrombosis in Users of non-Oral Hormonal Contraception: Follow-Up Study, Denmark 2001-10. BMJ (2012) 344:e2990. doi: 10.1136/bmj.e2990 22577198 PMC3349780

[15] Sweetland S Beral V Balkwill A Liu B Benson VS Canonico M . Venous Thromboembolism Risk in Relation to Use of Different Types of Postmenopausal Hormone Therapy in a Large Prospective Study. J Thromb Haemost (2012) 10(11):2277–86. doi: 10.1111/j.1538-7836.2012.04919.x 22963114

[16] Ruszkowska B Gadomska G Bielis L Gruszka M Góralczyk B Rość D . Risk of Venous Thromboembolic Disease in Postmenopausal Women Taking Oral or Transdermal Hormone Replacement Therapy. J Zhejiang Univ Sci B (2011) 12(1):12–7. doi: 10.1631/jzus.B1000106 PMC3017411 21194181

[17] Ott J Kaufmann U Bentz E-K Huber JC Tempfer CB . Incidence of Thrombophilia and Venous Thrombosis in Transsexuals Under Cross-Sex Hormone Therapy. Fertil Steril (2010) 93(4):1267–72. doi: 10.1016/j.fertnstert.2008.12.017 19200981

[18] Goldstein Z Khan M Reisman T Safer JD . Managing the Risk of Venous Thromboembolism in Transgender Adults Undergoing Hormone Therapy. J Blood Med (2019) 10:209–16. doi: 10.2147/JBM.S166780 PMC6628137 31372078

[19] van Adrichem RA Debeij J Nelissen RG Schipper IB Rosendaal FR Cannegieter SC . Below-Knee Cast Immobilization and the Risk of Venous Thrombosis: Results From a Large Population-Based Case-Control Study. J Thromb Haemost (2014) 12(9):1461–9. doi: 10.1111/jth.12655 25040873

[20] van Vlijmen EF Wiewel-Verschueren S Monster TB Meijer K . Combined Oral Contraceptives, Thrombophilia and the Risk of Venous Thromboembolism: A Systematic Review and Meta-Analysis. J Thromb Haemost (2016) 14(7):1393–403. doi: 10.1111/jth.13349 27121914

[21] Pomp ER le Cessie S Rosendaal FR Doggen CJ . Risk of Venous Thrombosis: Obesity and Its Joint Effect With Oral Contraceptive Use and Prothrombotic Mutations. Br j Haematol (2007) 139(2):289–96. doi: 10.1111/j.1365-2141.2007.06780.x 17897305

[22] Pomp ER Rosendaal FR Doggen CJ . Smoking Increases the Risk of Venous Thrombosis and Acts Synergistically With Oral Contraceptive Use. Am J Hematol (2008) 83(2):97–102. doi: 10.1002/ajh.21059 17726684

[23] Sandset PM . Mechanisms of Hormonal Therapy Related Thrombosis. Thromb Res (2013) 131 Suppl 1:S4–7. doi: 10.1016/S0049-3848(13)70009-4 23452740

[24] Kotamarti VS Greige N Heiman AJ Patel A Ricci JA . Risk for Venous Thromboembolism in Transgender Patients Undergoing Cross-Sex Hormone Treatment: A Systematic Review. J Sex Med (2021) 18(7):1280–91. doi: 10.1016/j.jsxm.2021.04.006 34140253

[25] Shamseer L Moher D Clarke M Ghersi D Liberati A Petticrew M . PRISMA-P Group. Preferred Reporting Items for Systematic Review and Meta-Analysis Protocols (PRISMA-P) 2015: Elaboration and Explanation. BMJ (2015) 350:g7647. doi: 10.1136/bmj.g7647 25555855

[26] Stroup DF Berlin JA Morton SC Olkin I Williamson GD Rennie D . Meta-Analysis of Observational Studies in Epidemiology: A Proposal for Reporting. Meta-Analysis Of Observational Studies in Epidemiology (MOOSE) Group. JAMA (2000) 283(15):2008–12. doi: 10.1001/jama.283.15.2008 10789670

[27] Bland M . Estimating Mean and Standard Deviation From the Sample Size, Three Quartiles, Minimum, and Maximum Estimating Mean and Standard Deviation From the Sample Size, Three Quartiles, Minimum, and Maximum. Int J Stat Med Res (2015) 4(1):57–64. doi: 10.6000/1929-6029.2015.04.01.6

[28] Hoy D Brooks P Woolf A Blyth F March L Bain C . Assessing Risk of Bias in Prevalence Studies: Modification of an Existing Tool and Evidence of Interrater Agreement. J Clin Epidemiol (2012) 65(9):934–39. doi: 10.1016/j.jclinepi.2011.11.014 22742910

[29] Higgins JP Thompson SG Deeks JJ Altman DG . Measuring Inconsistency in Meta-Analyses. BMJ (2003) 327(7414):557–60. doi: 10.1136/bmj.327.7414.557 PMC192859 12958120

[30] Sterne JA Egger M . Funnel Plots for Detecting Bias in Meta-Analysis: Guidelines on Choice of Axis. J Clin Epidemiol (2001) 54(10):1046–55. doi: 10.1016/s0895-4356(01)00377-8 11576817

[31] Begg CB Mazumdar M . Operating Characteristics of a Rank Correlation Test for Publication Bias. Biometrics (1994) 50(4):1088–101. doi: 10.2307/2533446 7786990

[32] Duval S Tweedie R . Trim and Fill: A Simple Funnel-Plot-Based Method of Testing and Adjusting for Publication Bias in Meta-Analysis. Biometrics (2000) 56(2):455–63. doi: 10.1111/j.0006-341x.2000.00455.x 10877304

[33] Arnold JD Sarkodie EP Coleman ME Goldstein DA . Incidence of Venous Thromboembolism in Transgender Women Receiving Oral Estradiol. J Sex Med (2016) 13(11):1773–7. doi: 10.1016/j.jsxm.2016.09.001 27671969

[34] Dittrich R Binder H Cupisti S Hoffmann I Beckmann MW Mueller A . Endocrine Treatment of Male-to-Female Transsexuals Using Gonadotropin-Releasing Hormone Agonist. Exp Clin Endocrinol Diabetes (2005) 113(10):586–92. doi: 10.1055/s-2005-865900 16320157

[35] Getahun D Nash R Flanders WD Baird TC Becerra-Culqui TA Cromwell L . Cross-Sex Hormones and Acute Cardiovascular Events in Transgender Persons: A Cohort Study. Ann Intern Med (2018) 169(4):205–13. doi: 10.7326/M17-2785 PMC6636681 29987313

[36] Jain J Kwan D Forcier M . Medroxyprogesterone Acetate in Gender-Affirming Therapy for Transwomen: Results From a Retrospective Study. J Clin Endocrinol Metab (2019) 104(11):5148–56. doi: 10.1210/jc.2018-02253 31127826

[37] Kozato A Fox GWC Yong PC Shin SJ Avanessian BK Ting J . No Venous Thromboembolism Increase Among Transgender Female Patients Remaining on Estrogen for Gender-Affirming Surgery. J Clin Endocrinol Metab (2021) 106(4):e1586–90. doi: 10.1210/clinem/dgaa966 33417686

[38] Meyer G Mayer M Mondorf A Flügel AK Herrmann E Bojunga J . Safety and Rapid Efficacy of Guideline-Based Gender-Affirming Hormone Therapy: An Analysis of 388 Individuals Diagnosed With Gender Dysphoria. Eur j Endocrinol (2020) 182(2):149–56. doi: 10.1530/EJE-19-0463 31751300

[39] Mullins ES Geer R Metcalf M Piccola J Lane A Conard LAE . Thrombosis Risk in Transgender Adolescents Receiving Gender-Affirming Hormone Therapy. Pediatrics (2021) 147(4):e2020023549. doi: 10.1542/peds.2020-023549 33753543

[40] Nolan IT Haley C Morrison SD Pannucci CJ Satterwhite T . Estrogen Continuation and Venous Thromboembolism in Penile Inversion Vaginoplasty. J Sex Me (2021) 18(1):193–200. doi: 10.1016/j.jsxm.2020.10.018 33243691

[41] Nota NM Wiepjes CM de Blok CJM Gooren LJG Kreukels BPC den Heijer M . Occurrence of Acute Cardiovascular Events in Transgender Individuals Receiving Hormone Therapy. Circulation (2019) 139(11):1461–2. doi: 10.1161/CIRCULATIONAHA.118.038584 30776252

[42] Prior JC Vigna YM Watson D . Spironolactone With Physiological Female Steroids for Presurgical Therapy of Male-to-Female Transsexualism. Arch Sex Behav (1989) 18(1):49–57. doi: 10.1007/BF01579291 2540730

[43] Pyra M Casimiro I Rusie L Ross N Blum C Keglovitz Baker K . An Observational Study of Hypertension and Thromboembolism Among Transgender Patients Using Gender-Affirming Hormone Therapy. Transgend Health (2020) 5(1):1–9. doi: 10.1089/trgh.2019.0061 32322683 PMC7173689

[44] Schlatterer K Yassouridis A von Werder K Poland D Kemper J . Stalla GK. A Follow-Up Study for Estimating the Effectiveness of a Cross-Gender Hormone Substitution Therapy on Transsexual Patients. Arch Sex Behav (1998) 27(5):475–92. doi: 10.1023/a:1018704630036 9795728

[45] Seal LJ Franklin S Richards C Shishkareva A Sinclaire C Barrett J . Predictive Markers for Mammoplasty and a Comparison of Side Effect Profiles in Transwomen Taking Various Hormonal Regimens. J Clin Endocrinol Metab (2012) 97(12):4422–8. doi: 10.1210/jc.2012-2030 23055547

[46] Tack LJW Heyse R Craen M Dhondt K Bossche HV Laridaen J . Consecutive Cyproterone Acetate and Estradiol Treatment in Late-Pubertal Transgender Female Adolescents. J Sex Med (2017) 14(5):747–57. doi: 10.1016/j.jsxm.2017.03.251 28499525

[47] van Kesteren PJ Asscheman H Megens JA Gooren LJ . Mortality and Morbidity in Transsexual Subjects Treated With Cross-Sex Hormones. Clin Endocrinol (Oxf) (1997) 47(3):337–42. doi: 10.1046/j.1365-2265.1997.2601068.x 9373456

[48] Wierckx K Elaut E Declercq E Heylens G De Cuypere G Taes Y . Prevalence of Cardiovascular Disease and Cancer During Cross-Sex Hormone Therapy in a Large Cohort of Trans Persons: A Case-Control Study. Eur J Endocrinol (2013) 169(4):471–8. doi: 10.1530/EJE-13-0493 23904280

[49] Wilson R Spiers A Ewan J Johnson P Jenkins C Carr S . Effects of High Dose Oestrogen Therapy on Circulating Inflammatory Markers. Maturitas (2009) 62(3):281–6. doi: 10.1016/j.maturitas.2009.01.009 19231116

[50] Asscheman H Gooren LJ Eklund PL . Mortality and Morbidity in Transsexual Patients With Cross-Gender Hormone Treatment. Metabolism (1989) 38(9):869–73. doi: 10.1016/0026-0495(89)90233-3 2528051

[51] Asscheman H Giltay EJ Megens JA de Ronde WP van Trotsenburg MA Gooren LJ . A Long-Term Follow-Up Study of Mortality in Transsexuals Receiving Treatment With Cross-Sex Hormones. Eur j Endocrinol (2011) 164(4):635–42. doi: 10.1530/EJE-10-1038 21266549

[52] Wierckx K Mueller S Weyers S Van Caenegem E Roef G Heylens G . Long-Term Evaluation of Cross-Sex Hormone Treatment in Transsexual Persons. J Sex Med (2012) 9(10):2641–51. doi: 10.1111/j.1743-6109.2012.02876.x 22906135

[53] Wierckx K Van Caenegem E Schreiner T Haraldsen I Fisher AD Toye K . Cross-Sex Hormone Therapy in Trans Persons is Safe and Effective at Short-Time Follow-Up: Results From the European Network for the Investigation of Gender Incongruence. J Sex Med (2014) 11(8):1999–2011. doi: 10.1111/jsm.12571 24828032

[54] Vandenbroucke JP Koster T Briet E Reitsma PH Bertina RM Rosendaal FR . Increased Risk of Venous Thrombosis in Oral-Contraceptive Users Who are Carriers of Factor V Leiden Mutation. Lancet (1994) 344(8935):1453–7. doi: 10.1016/s0140-6736(94)90286-0 7968118

[55] Toorians A Thomassen M Zweegman S Magdeleyns EJ Tans G Gooren LJ . Venous Thrombosis and Changes of Hemostatic Variables During Cross-Sex Hormone Treatment in Transsexual People. J Clin Endocrinol Metab (2003) 88(12):5723–9. doi: 10.1210/jc.2003-030520 14671159

[56] Sitruk-Ware R Nath A . Characteristics and Metabolic Effects of Estrogen and Progestins Contained in Oral Contraceptive Pills. Best Pract Res Clin Endocrinol Metab (2013) 27(1):13–24. doi: 10.1016/j.beem.2012.09.004 23384742

