# Supplementary figures and images for: Risk of Venous Thromboembolism in Transgender People Undergoing Hormone Feminizing Therapy: A Prevalence Meta-Analysis and Meta-Regression Study
Source: Front Endocrinol (Lausanne). 2021 Nov 9;12:741866. doi: 10.3389/fendo.2021.741866 (PMC8647165; doi:10.3389/fendo.2021.741866)

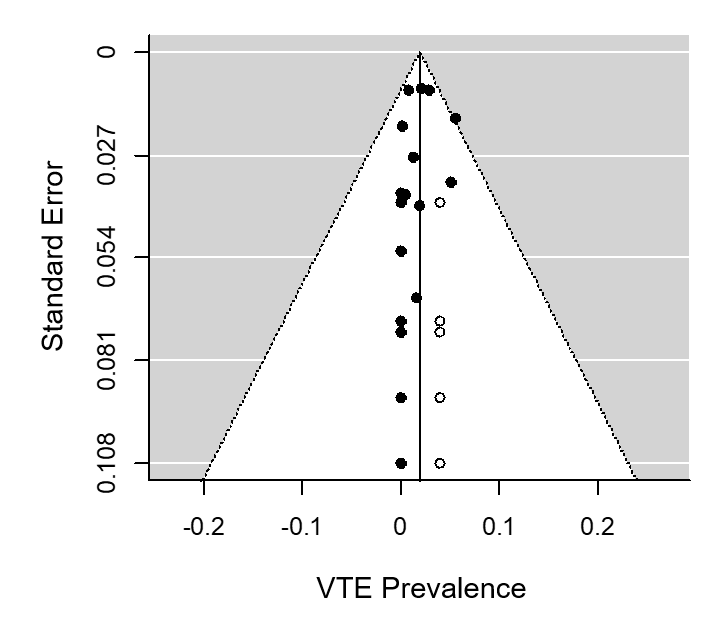

Supplement: Supplementary Figure 1 — Funnel plots of results from studies assessing the prevalence of venous thromboembolism (VTE). The trim-and-fill analysis identified five putative missing studies (white circle) on the right side of distribution. [file Image_1.tif]
