# Supplementary material for: Risk of Venous Thromboembolism in Transgender People Undergoing Hormone Feminizing Therapy: A Prevalence Meta-Analysis and Meta-Regression Study
Source: Front Endocrinol (Lausanne). 2021 Nov 9;12:741866. doi: 10.3389/fendo.2021.741866 (PMC8647165; doi:10.3389/fendo.2021.741866)
Supplement: Supplementary file 2 [file Table_1.doc]

**Supplementary Table 1A.** PRISMA-P (Preferred Reporting Items for Systematic review and Meta-Analysis Protocols) 2015 checklist: recommended items to address in a systematic review protocol

| Section and topic | Item No | Checklist item | Section |
| --- | --- | --- | --- |
| ADMINISTRATIVE INFORMATION | | |  |
| Title: |  |  |  |
| Identification | 1a | Identify the report as a protocol of a systematic review | Title |
| Update | 1b | If the protocol is for an update of a previous systematic review, identify as such |  |
| Registration | 2 | If registered, provide the name of the registry (such as PROSPERO) and registration number | Methods: PROSPERO registration number: CRD42021229916 |
| Authors: |  |  |  |
| Contact | 3a | Provide name, institutional affiliation, e-mail address of all protocol authors; provide physical mailing address of corresponding author | Title page |
| Contributions | 3b | Describe contributions of protocol authors and identify the guarantor of the review | Section: author contributions |
| Amendments | 4 | If the protocol represents an amendment of a previously completed or published protocol, identify as such and list changes; otherwise, state plan for documenting important protocol amendments | NA |
| Support: |  |  |  |
| Sources | 5a | Indicate sources of financial or other support for the review | NA |
| Sponsor | 5b | Provide name for the review funder and/or sponsor | NA |
| Role of sponsor or funder | 5c | Describe roles of funder(s), sponsor(s), and/or institution(s), if any, in developing the protocol | NA |
| INTRODUCTION | | |  |
| Rationale | 6 | Describe the rationale for the review in the context of what is already known | Introduction |
| Objectives | 7 | Provide an explicit statement of the question(s) the review will address with reference to participants, interventions, comparators, and outcomes (PECOS) | Introduction (aim) and supplementary Table 2 (PECOS) |
| METHODS | | |  |
| Eligibility criteria | 8 | Specify the study characteristics (such as PICO, study design, setting, time frame) and report characteristics (such as years considered, language, publication status) to be used as criteria for eligibility for the review | Section: ‘Inclusion and exclusion criteria’ in methods and PECOS in supplementary Table 2 |
| Information sources | 9 | Describe all intended information sources (such as electronic databases, contact with study authors, trial registers or other grey literature sources) with planned dates of coverage | Section: ‘Systematic search strategy’ in methods |
| Search strategy | 10 | Present draft of search strategy to be used for at least one electronic database, including planned limits, such that it could be repeated | Section: ‘Systematic search strategy’ in methods and Figure 1 (PRISMA flowchart) |
| Study records: |  |  |  |
| Data management | 11a | Describe the mechanism(s) that will be used to manage records and data throughout the review | Sections: ‘Systematic search strategy’, ‘Inclusion and exclusion criteria’ and ‘data extraction’ in methods. |
| Selection process | 11b | State the process that will be used for selecting studies (such as two independent reviewers) through each phase of the review (that is, screening, eligibility and inclusion in meta-analysis) | Sections: ‘Systematic search strategy’ and ‘Inclusion and exclusion criteria’ |
| Data collection process | 11c | Describe planned method of extracting data from reports (such as piloting forms, done independently, in duplicate), any processes for obtaining and confirming data from investigators | Section: ‘data extraction’ in methods. |
| Data items | 12 | List and define all variables for which data will be sought (such as PICO items, funding sources), any pre-planned data assumptions and simplifications | Supplementary Table 2 (PECOS) |
| Outcomes and prioritization | 13 | List and define all outcomes for which data will be sought, including prioritization of main and additional outcomes, with rationale | Introduction (aim), supplementary Table 2 (PECOS) and Section ‘Inclusion and exclusion criteria’ in methods |
| Risk of bias in individual studies | 14 | Describe anticipated methods for assessing risk of bias of individual studies, including whether this will be done at the outcome or study level, or both; state how this information will be used in data synthesis | Sections: ‘quality assessment’ and ‘statistical analysis’ in methods. Table 2. |
| Data synthesis | 15a | Describe criteria under which study data will be quantitatively synthesised | Statistical analysis in methods. Figure 2, Figure 4A and B, Figure 5A and B |
|  | 15b | If data are appropriate for quantitative synthesis, describe planned summary measures, methods of handling data and methods of combining data from studies, including any planned exploration of consistency (such as I^2^, Kendall’s τ) | Statistical analysis in methods. Figure 2, Figure 4A and B, Figure 5A and B |
|  | 15c | Describe any proposed additional analyses (such as sensitivity or subgroup analyses, meta-regression) | Statistical analysis in methods. Figure 3 (meta-regressions), Figure 4A and B 5A and B (subgroup analysis) |
|  | 15d | If quantitative synthesis is not appropriate, describe the type of summary planned | Statistical analysis in methods. Figure 2 |
| Meta-bias(es) | 16 | Specify any planned assessment of meta-bias(es) (such as publication bias across studies, selective reporting within studies) | Statistical analysis in methods. Supplementary Figure 1 (funnel plots and trim and fill analysis) |
| Confidence in cumulative evidence | 17 | Describe how the strength of the body of evidence will be assessed (such as GRADE) | Section quality assessment in methods. Table 2. Discussion. |

*From: Shamseer L, Moher D, Clarke M, Ghersi D, Liberati A, Petticrew M, Shekelle P, Stewart L, PRISMA-P Group. Preferred reporting items for systematic review and meta-analysis protocols (PRISMA-P) 2015: elaboration and explanation. BMJ. 2015 Jan 2;349(jan02 1):g7647.*

**Supplementary Table 1B. MOOSE Checklist for Meta-analyses of Observational Studies**

| **Item No** | **Recommendation** | **Reported section** |
| --- | --- | --- |
| Reporting of background should include | | |
| 1 | Problem definition | Introduction |
| 2 | Hypothesis statement | Introduction |
| 3 | Description of study outcome(s) | Introduction/Methods |
| 4 | Type of exposure or intervention used | Methods |
| 5 | Type of study designs used | Methods |
| 6 | Study population | Methods: Inclusion and exclusion criteria |
| Reporting of search strategy should include | | |
| 7 | Qualifications of searchers (eg, librarians and investigators) | Methods |
| 8 | Search strategy, including time period included in the synthesis and key words | Methods: Systematic search strategy |
| 9 | Effort to include all available studies, including contact with authors | Methods: Systematic search strategy and  data extraction |
| 10 | Databases and registries searched | Methods: Systematic search strategy |
| 11 | Search software used, name and version, including special features used (eg, explosion) | Statistical analysis |
| 12 | Use of hand searching (eg, reference lists of obtained articles) | Figure 1 |
| 13 | List of citations located and those excluded, including justification | Study selection in Results and Figure 1 |
| 14 | Method of addressing articles published in languages other than English | NA |
| 15 | Method of handling abstracts and unpublished studies | NA |
| 16 | Description of any contact with authors | Data extraction |
| Reporting of methods should include | | |
| 17 | Description of relevance or appropriateness of studies assembled for assessing the hypothesis to be tested | Methods: Quality assessment |
| 18 | Rationale for the selection and coding of data (eg, sound clinical principles or convenience) | Methods: Inclusion and exclusion criteria |
| 19 | Documentation of how data were classified and coded (eg, multiple raters, blinding and interrater reliability) | Methods: Quality assessment |
| 20 | Assessment of confounding (eg, comparability of cases and controls in studies where appropriate) | Methods: Statistical analysis |
| 21 | Assessment of study quality, including blinding of quality assessors, stratification or regression on possible predictors of study results | Methods: Quality assessment |
| 22 | Assessment of heterogeneity | Methods: Statistical analysis; Results: Meta-regression and sub-group analysis, Figure 3; Figure 4A and B, Figure 5A and B |
| 23 | Description of statistical methods (eg, complete description of fixed or random effects models, justification of whether the chosen models account for predictors of study results, dose-response models, or cumulative meta-analysis) in sufficient detail to be replicated | Methods: Statistical analysis |
| 24 | Provision of appropriate tables and graphics | Table 1, Table 2, Supplementary Table 2; Supplementary Table 3; Figures 1-5; Supplementary Figure 1 |
| Reporting of results should include | | |
| 25 | Graphic summarizing individual study estimates and overall estimate | Synthesis of results: Figure 2 |
| 26 | Table giving descriptive information for each study included | Table 1 |
| 27 | Results of sensitivity testing (eg, subgroup analysis) | Figure 4A and B, Figure 5A and B |
| 28 | Indication of statistical uncertainty of findings | Table 1 and 2 |
| Reporting of discussion should include | | |
| 29 | Quantitative assessment of bias (eg, publication bias) | Supplementary Figure 1 |
| 30 | Justification for exclusion (eg, exclusion of non-English language citations) | Figure 1 |
| 31 | Assessment of quality of included studies | Results: Quality of the included studies and Table 2 |
| Reporting of conclusions should include | | |
| 32 | Consideration of alternative explanations for observed results | Discussion |
| 33 | Generalization of the conclusions (ie, appropriate for the data presented and within the domain of the literature review) | Discussion |
| 34 | Guidelines for future research | Discussion |
| 35 | Disclosure of funding source | NA |

*From*: Stroup DF, Berlin JA, Morton SC, et al, for the Meta-analysis Of Observational Studies in Epidemiology (MOOSE) Group. Meta-analysis of Observational Studies in Epidemiology. A Proposal for Reporting. *JAMA*. 2000;283(15):2008-2012. doi: 10.1001/jama.283.15.2008.
