# Supplementary material for: Risk of Venous Thromboembolism in Transgender People Undergoing Hormone Feminizing Therapy: A Prevalence Meta-Analysis and Meta-Regression Study
Source: Front Endocrinol (Lausanne). 2021 Nov 9;12:741866. doi: 10.3389/fendo.2021.741866 (PMC8647165; doi:10.3389/fendo.2021.741866)
Supplement: Supplementary file 3 [file Table_2.docx]

**Supplementary Table 2.** PECOS model

| **P**articipants: | Assigned Males at Birth (AMAB) trans people undergoing hormone feminizing therapy recruited from the general population or from cohorts of patients |
| --- | --- |
| **E**xposure: | Any diagnosis of venous thrombo-embolism |
| **C**omparator/**C**ontrol: | Not applicable |
| **O**utcomes: | Prevalence of venous thrombo-embolism |
| **S**tudy design: | Any (prior systematic reviews and meta-analyses were not included but they were screened for individual studies) |
