# Supplementary material for: Risk of Venous Thromboembolism in Transgender People Undergoing Hormone Feminizing Therapy: A Prevalence Meta-Analysis and Meta-Regression Study
Source: Front Endocrinol (Lausanne). 2021 Nov 9;12:741866. doi: 10.3389/fendo.2021.741866 (PMC8647165; doi:10.3389/fendo.2021.741866)
Supplement: Supplementary file 4 [file Table_3.docx]

**Supplementary Table 3.** Additional characteristics of the included studies

| **Study** | **AMAB**  **(n)** | **VTE**  **(n, %)** | **Administration** | **Type of estrogen** | **Estrogen dosing regimen** | **Oral estradiol**  **(n, %)** | **Spironolactone (n, %)** | **Ciproterone acetato**  **(n, %)** | **Progesterone (n, %)** | **GnRH AG/ANT**  **(n, %)** | **Finasteride (n, %)** | **Surgery**  **(n, %)** |
| --- | --- | --- | --- | --- | --- | --- | --- | --- | --- | --- | --- | --- |
| Arnold et al., 2016 (33) | 676 | 1 (0.15%) | oral | E | NR | 676 (100%) | 634 (93.8%) | 0 (0.0%) | 27 (4.0%) | 0 (0.0%) | 112 (16.6%) | NR |
| Dittrich et al., 2005 (34) | 60 | 1 (1.67%) | oral | EV | 6 mg QD | 60 (100%) | 0 (0.0%) | 0 (0.0%) | 0 (0.0%) | 60 (100%) | 0 (0.0%) | NR |
| Getahun et al., 2018 (35) | 2842 | 61 (2.15%) | NR | E | NR | 853(30.0%) | NR | NR | NR | NR | NR | NR |
| Jain et al., 2019 (36) | 92 | 0 (0.00%) | sublingual | E | 2 to 12 mg QD | 0 (0.0%) | 92 (100%) | 0 (0.0%) | 39 (42.4%) | 0 (0.0%) | 0 (0.0%) | NR |
| Kozato et al., 2021 (37) | 662 | 1 (0.20%) | oral or trandermal or injectable | E | OE 5.0 mg QD;  IE 54 mg PM | 210 (31.7%) | NR | NR | NR | NR | NR | 662 (100%) |
| Meyer et al., 2020 (38) | 155 | 3 (1.90%) | oral or transdermal | EV or EH | NR | 73(47.1%) | NR | NR | NR | NR | NR | NR |
| Mullins et al., 2021 (39) | 182 | 0 (0.00%) | oral or transdermal or intramuscular | E | 4 mg QD | 165(90.7%) | 0 (0.0%) | 0 (0.0%) | 0 (0.0%) | NR | NR | NR |
| Nolan et al., 2021 (40) | 178 | 1 (0.60%) | oral or transdermal | estrogen | NR | NR | 0 (0.0%) | 0 (0.0%) | NR | 0 (0.0%) | 0 (0.0%) | 178 (100%) |
| Nota et al., 2019 (41) | 2517 | 73 (2.90%) | NR | NR | NR | NR | NR | NR | NR | NR | NR | NR |
| Ott et al., 2010 (17) | 162 | 0 (0.00%) | transdermal | E_2_ | 0.2 mg PW | 0 (0.0%) | 0 (0.0%) | NR | 0 (0.0%) | 0 (0.0%) | NR | 24 (14.8%) |
| Pyra et al., 2020 (43) | 2509 | 19 (0.80%) | oral or patch or injectable or implantor vaginal | estrogen | NR | 946 (37.7%) | NR | NR | 354 (14.1%) | NR | 1530 (61.0%) | NR |
| Prior et al., 1989 (42) | 50 | 0 (0.00%) | oral | CEE | 2.5 mg BD | 50 (100%) | 50 (100%) | 0 (0.0%) | 0 (0.0%) | 0 (0%) | 0 (0.0%) | NR |
| Schlatterer et al., 1998 (44) | 46 | 0 (0.00%) | oral (56.5%) or intramuscular (37.0%) | E or E_2_ | 40-100 mg | 26 (56.5%) | NR | 26 (56.5%) | NR | 0 (0.0%) | 0 (0%) | 24 (52.2%) |
| Seal et al., 2012 (45) | 330 | 4 (1.20%) | oral | EV (49.4%) or EE (40.3%) or CEE (10.9%) | NR | 330 (100%) | 22 (6.7%) | 82 (24.8%) | NR | 91 (27.6%) | 48 (14.5%) | NR |
|  |  |  |  |  |  |  |  |  |  |  |  |  |
| Tack et al., 2017 (46) | 21 | 0 (0.00%) | oral | EV | 0.5-2 mg QD | 21 (100%) | 0 (0.0%) | 21 (100%) | 0 (0.0%) | 0 (0.0%) | 0 (0.0%) | NR |
| van Kesteren et al., 1997 (47) | 816 | 45 (5.50%) | oral or transdermal (16.9%) | EE | Oral: 100 mg QD, trasdermal: 100 o 50 mg QD | NR | NR | 816 (100%) | NR | NR | NR | NR |
| Wierckx et al., 2013 (48) | 214 | 11 (5.10%) | oral (46.3%) or transdermal (49.0%) | EV (42.5%) | E_2_ gel 1.5 mg QD (35.5%); E patch 50 mg QD (13.6%); EV 2 mg QD (42.5%); estriol 2 mg QD (0.4%);  EE 50 mg QD (0.9%);  EE OC 30–50 mg QD (2.3%) | 99 (46.3%) | NR | NR | NR | NR | NR | 129 (64.8%) |
| Wilson et al., 2009 (49) | 30 | 0 (0.00%) | oral or transdermal | E | From 1.25 mg QD to  2.5 mg QD | 23 (76.7%) | NR | NR | NR | NR | NR | NR |
|  |  |  |  |  |  |  |  |  |  |  |  |  |
| Values are presented as mean or number (%). Abbreviations: AG, agonist; AMAB, Assigned Males at Birth; ANT, antagonist; BD, twice a day; CEE, conjugated equine estrogens; E_2_, 17β-estradiol; E, estradiol; EE, ethinyl estradiol; EH, estradiol hemihydrate; EV, estradiol valerate; GnRH, Gonadotropin Releasing Hormone; IE, injectable estradiol; NR, not reported; OC, oral contraceptive; OE, oral estradiol; QD, once a day; PM, per month; PW, per week; VTE, venous thromboembolic events. | | | | | | | | | | | | |
